# MiR-424-3p suppresses galectin-3 expression and sensitizes ovarian cancer cells to cisplatin

**DOI:** 10.1007/s00404-018-4999-7

**Published:** 2018-12-26

**Authors:** Dominik Bieg, Daniel Sypniewski, Ewa Nowak, Ilona Bednarek

**Affiliations:** Department of Biotechnology and Genetic Engineering, School of Pharmacy with the Division of Laboratory Medicine in Sosnowiec, Medical University of Silesia, Katowice, Poland, Jedności 8 Street, 41-200 Sosnowiec, Poland

**Keywords:** Ovarian cancer, Drug resistance, Cisplatin, Galectin-3, miR-424-3p

## Abstract

**Purpose:**

Assessment of miR-424-3p mimic capability to sensitize SK-OV-3 and TOV-21G ovarian cancer cells to cisplatin by decreasing the expression of galectin-3, which is an anti-apoptotic protein overexpressed in ovarian cancer and associated with resistance to chemotherapy.

**Methods:**

We performed a reverse transfection of miR-424-3p mimic into SK-OV-3 and TOV-21G ovarian cancer cells, followed by Real Time™ RT-PCR analysis of the expression of miR-424-3p and galectin-3 mRNA as well as ELISA assay for galectin-3 protein level. Next, we studied the viability (XTT assay), proliferation (EdU incorporation assay), and apoptosis (ELISA assay) of the both cell lines transfected with the mimic and treated with cisplatin.

**Results:**

We demonstrated that miR-424-3p mimic effectively transfects into SK-OV-3 and TOV-21G ovarian cancer cells in which it significantly suppresses the expression of galectin-3 at the protein level, but not at the mRNA level. Reverse transfection of both cell lines with the mimic, followed by treatment with cisplatin, resulted in a reduction in cell viability and proliferation as well as an increase in the induction of apoptosis.

**Conclusions:**

MiR-424-3p mimic sensitizes SK-OV-3 and TOV-21G ovarian cancer cells to cisplatin by decreasing the expression of galectin-3.

## Introduction

Ovarian cancer is the fifth leading cause of cancer death among women worldwide, and by that, it is considered to be one of the most deadly gynecological cancers [1–3]. The 5-year survival rate of the cancer ranges from approximately 20% to 90%. The prognosis are mostly successful in the case of its early detection; however, in most cases, patients are not diagnosed at the early stages of the disease [1, 4]. Standard treatment for advanced ovarian cancer is surgery combined with platinum- and taxane-based therapy. Initially, the clinical response rate after chemotherapy is often high, but frequently occurring relapses lead to an acquired resistance to the anticancer drugs and, as a consequence, most patients ultimately die [1–3]. One of the proteins, associated with poor survival rates as well as chemoresistance in ovarian cancer, is galectin-3 [3, 5].

Galectin-3 (coded by *LGALS3* gene) is a lectin-containing an N-terminal domain that regulates its cellular function, an α-collagen-like sequence, and a C-terminal carbohydrate recognition domain (CRD) that binds to β-galactosides [3, 6]. The C-terminal domain also includes the NWGR (Asp–Trp–Gly–Arg) anti-death motif, which is a part of highly conserved BH1 domain specific to the BCL-2 protein family. The BH1 domain is critical for an anti-apoptotic activity of BCL-2 and also allows cytoplasmic galectin-3 to function as an anti-apoptotic molecule [6, 7]. The NWGR anti-death motif suppresses apoptosis of cancer cells induced by chemotherapeutic agents, such as cisplatin, and thus, galectin-3 plays an important role in the resistance to anticancer drugs [6, 8]. Increasing evidence suggests that galectin-3 promotes chemoresistance in prostate cancer, cholangiocarcinoma, thyroid carcinoma [9], lung cancer [5], and ovarian cancer [3, 5] as well as protects BT549 human breast carcinoma cells from apoptosis induced by cisplatin, anthracycline, adriamycin, and 5-FU (5-fluorouracil) [5]. Moreover, overexpression of galectin-3 has been reported in multiple types of human tumors, including: ovarian cancer [7]; pancreatic cancer [5, 6, 10, 11]; breast cancer [5, 10, 11]; thyroid, gastric and colon cancer [5, 7, 11]; hepatocellular, head and neck, prostate cancer, and glioma [10]; T lymphoma, Burkitt lymphoma, and cervical cancer [11]. Furthermore, despite contribution in drug resistance, galectin-3 also exhibits pleiotropic biological functions, and is also involved in various pathological cellular processes, such as malignant: transformation, adhesion, angiogenesis, migration, and metastasis [6, 10]. Therefore, downregulation of galectin-3 expression in ovarian neoplasm may be an effective anticancer strategy [8], and could be achieved, for example, by specific microRNA mimic molecules [9, 12].

MicroRNAs (miRNAs) are small, non-coding RNAs of 18–25 nucleotides (approximately 22-nucleotide), highly conserved among a wide range of species and abled to posttranscriptional gene regulation. miRNAs control at least 30% of protein-coding human genes and are irregularly expressed in many neoplasms, including ovarian cancer in which they are involved in various cellular functions, such as cell cycle, proliferation, apoptosis, invasion, progression, and metastasis [1, 4]. Increasing evidence also suggests that miRNAs are engaged in the modulation of pathways associated with drug resistance of some tumors, including ovarian cancer [1, 4, 13–15]. MiRNAs negatively regulate genes expression by binding their 5′ seed region (in general, nucleotides 2–8 of the miRNA) to the 3′UTR (untranslated region) of target mRNAs [1].

Ramasamy et al. [12] discovered that galectin-3 mRNA 3′UTR contains target sequence for miRNA molecule, which they named as miR-322, due to its similarity to the sequence of mouse and rat. Furthermore, they proved that this miRNA can modulate galectin-3 expression by decreasing its level. They also claimed that miR-322 and miR-424 are two mature strands resulting from the cleavage of a common pre-miRNA stem-loop structure [12]. According today’s knowledge and nomenclature, the pre-miRNA stem-loop structure gives not one, but two functional mature strands: guide (from 5′ end, marked as -5p) and passenger (from 3′ end, marked as -3p). Moreover, mouse and rat pre-miR-322 stem-loop structure is the ortholog of human pre-miR-424 stem-loop structure and both of them give two mature miRNA strands [16, 17]. Therefore, the miRNA molecule studied by Ramasamy et al. [12] is actually miR-424-3p and it is expressed in human cells. Furthermore, there is an evidence that miR-424-3p is capable of sensitizing chemoresistant non-small-cell lung cancer cells to cisplatin and paclitaxel [13]. In this place, it should be noticed that searching any data about miR-424-3p must be very careful and must include sequence comparison (if it is available), because of the fact that most of the papers do not clearly specify the exact subtype of miR-424, which was the subject of study. Moreover, although miR-424-5p and miR-424-3p are from the same origin, they could have quite opposite effect on cells functioning, what was confirmed in hepatocellular and pancreatic cancers [13].

In view of the fact that miR-424-3p can reduce the expression of galectin-3 (the anti-apoptotic protein associated with cancers chemoresistance) and, on the other hand, sensitize chemoresistant non-small-cell lung cancer cells to cisplatin, we hypothesized that transfection of miR-424-3p which mimic into ovarian cancer cells will sensitize them to cisplatin, what will be achieved by reducing the expression of galectin-3.

## Materials and methods

### Cell culture, reverse transfection, and apoptosis induction

Human ovarian cancer cells were purchased from the American Type Culture Collection (ATCC) and cultured in a humidified atmosphere of 95% air and 5% CO_2_ at 37 °C. TOV-21G cells (cisplatin-sensitive; grade 3, stage III, primary malignant adenocarcinoma; clear cell carcinoma; ATCC^®^ CRL-11730™) were cultivated in the mixture (1:1) of M-199 Earle’s Salts Base medium (Biological Industries) and MCDB-105 medium (Biological Industries) supplemented with 15% (v/v) FBS (Gibco^®^) and gentamicin (Biological Industries) at the final concentration of 50 µg/ml. SK-OV-3 cells (cisplatin-resistant; adenocarcinoma; ATCC^®^ HTB-77™) were grown in RPMI-1640 medium (Lonza) supplemented with 10% (v/v) FBS (fetal bovine serum; Gibco^®^) and gentamicin (Biological Industries) at the final concentration of 50 µg/ml.

RNA oligonucleotides used in this study were: hsa-miR-424-3p miRCURY LNA™ miRNA mimic (QIAGEN) labeled with 5′-FAM and imitating mature, human miR-424-3p miRNA (5′-CAAAACGUGAGGCGCUGCUAU-3′); the negative control included in Silencer^®^ siRNA Transfection II Kit (Ambion^®^), which is a scrambled sequence bearing no homology to the human genome.

Lipofectamine^®^ 2000 Reagent (Invitrogen™) was used for the reverse transfection of the RNA oligonucleotides into cells according to the manufacturer’s protocol (the procedure described below was performed in case of 96-well plates, and the amount of reagents for other plates was proportionally increased, e.g., fivefold for 24-well plates). Briefly, the addition of gentamicin to the growth media was removed on the day before the reverse transfection and this limitation was maintained until the end of each individual experiment. Lipofectamine^®^ 2000 Reagent was diluted in 25 µl Opti-MEM^®^ I (1X) Reduced Serum Medium with GlutaMAX™ (Gibco^®^) for the final concentration of 5 µg/ml in the reverse transfection, incubated for 5 min at room temperature, and added to the RNA oligonucleotides diluted in 25 µl Opti-MEM^®^ I (1X) Reduced Serum Medium with GlutaMAX™ (Gibco^®^) for the final concentration of 25 nM in the reverse transfection. After 15 min incubation at room temperature, 100 μl of complete growth medium without antibiotics and with 1.6 × 10^4^ cells was added to each well containing RNA molecule-Lipofectamine^®^ 2000 Reagent complex. After 24 h incubation in the standard condition (5% CO_2_, 37 °C), the reverse transfection mixtures were removed from wells and replaced with fresh serum-free and antibiotics-free media. The reverse transfection efficiency was assessed by counting the ratio of cells successfully transfected with 5′-FAM-labeled miR-424-3p mimic (the presence of green fluorescence) to the total number of cells (in three different fields) using an Eclipse Ti (Nikon Instruments Inc.) fluorescence microscope.

Cisplatin was acquired from Sigma-Aldrich, dissolved in 0.9% NaCl solution (Polpharma) at the concentration of 1 mg/ml (3.333 mM), and stored in − 20 °C. The cells were treated with cisplatin 48 h after the reverse transfection (for another 24 h) to induce apoptosis.

### RNA extraction

Total RNA was isolated from cells reverse transfected with RNA oligonucleotides in 24-well plates (24 h, 48 h, and 72 h after reverse transfection) using TRI Reagent™ Solution (Invitrogen™) and Direct-zol™ RNA MiniPrep (ZymoResearch) according to the manufacturer’s protocol. The concentration and purity of extracts were measured spectrophotometrically by BioPhotometer (Eppendorf) supplemented with µCuvette^®^ G1.0 (Eppendorf). Quality and integrity of isolated RNA were also verified by 1% agarose gel electrophoresis.

### Real Time™ stem-loop RT-PCR analysis

Total RNA extracts were reverse transcribed with MMLV High-Performance Reverse Transcriptase (Epicentre^®^) and one of the specific primers: (1st) stem–loop RT primer (5′-GTC GTA TCC AGT GCA GGG TCC GAG GTA TTC GCA CTG GAT ACG ACA TAG CAG C-3′) specific for miR-424-3p and designed by Huang et al. [18] or (2nd) reverse primer (5′-GCC AGT CTG GAC TGT TCT TCA-3′) specific for *TBP* (*TATA*-*box-binding protein*) housekeeping gene, which was chosen as a reference gene for normalization. The reverse primer was designed using the *Primer*-*BLAST* software (NCBI). A single reverse transcription reaction mixture contained: 1× Reaction Buffer; 10 mM DTT; 0.5 mM dNTPs; 12.5 nM miR-424-3p stem-loop RT primer or 1 nM *TBP* reverse primer; 250 ng of total cell RNA; 25 U of MMLV High-Performance Reverse Transcriptase; ddH_2_O supplemented to the final volume of 10 µl. The samples were incubated in the T100™ Thermal Cycler (Bio-Rad) at 16 °C for 10 min, 42 °C for 60 min, and 85 °C for 5 min.

Quantitative PCR (qPCR) was performed using the Stratagene Mx3000P Instrument and ZymoTaq™ PreMix (Zymo Research) supplemented with SYBR^®^ Green I Nucleic Acid Gel Stain (Invitrogen™) and ROX (Agilent Technologies). The miR-424-3p specific primers for qPCR reaction were designed by Huang et al. [18] and had the following sequences: 5′-CGC AAA ACG TGA GGC GCT-3′ (forward) and 5′-CCA GTG CAG GGT CCG AGG TA-3′ (reverse). The *TBP*-specific primers for qPCR reaction were designed using the *Primer*-*BLAST* software (NCBI) and had the sequences as followed: 5′-TAT AAT CCC AAG CGG TTT GCT G-3′ (forward) and 5′-GCC AGT CTG GAC TGT TCT TCA-3′ (reverse). Each of the qPCR reaction mixtures consisted of: 1× ZymoTaq™ PreMix; 0.075× SYBR^®^ Green; 30 nM ROX; 0.6 µM forward and reverse primers; 0.5 µl of cDNA solution from reverse transcription and ddH_2_O supplemented to the final volume of 12.5 µl. The thermal profile of qPCR reaction was as follows: initial denaturation (95 °C, 10 min); 40 cycles of denaturation (95 °C, 30 s), annealing (60 °C, 30 s, fluorescence measurement at the endpoint), and extension (72 °C, 30 s); final extension (72 °C, 7 min). The specificity of amplification products were assessed by melting temperature analysis and 3% agarose gel electrophoresis. The expression of miR-424-3p was quantified using the comparative $\left( { 2^{{ - \Delta \Delta C_{\text{q}} }} } \right)$ method. All cell groups were compared against untransfected cells control group at 24 h after the reverse transfection, considered as 100%. Samples were prepared in triplicate and Real Time™ RT-PCR measurement for each sample was done in duplicate.

### Real Time™ RT-PCR analysis

The Real Time™ RT-PCR was performed using Brilliant II SYBR^®^ Green QRT-PCR Master Mix Kit (Agilent Technologies). A single reaction mixture was prepared according to the manufacturer’s protocol and contained: 1× Master Mix; 30 nM ROX; 0.2 µM forward and reverse primers; 100 ng of total cell RNA; 0.6 µl RT/RNase Block Enzyme Mixture and ddH_2_O supplemented to the final volume of 15 µl. The analysis was conducted on the Stratagene Mx3000P Instrument according to the thermal profile, which was as follows: reverse transcription (55 °C, 30 min); initial denaturation (95 °C, 10 min); 40 cycles of denaturation (94 °C, 15 s), annealing (63 °C, 60 s, fluorescence measurement at the endpoint) and extension (72 °C, 30 s); final extension (72 °C, 10 min). The amplification products specificity was confirmed by the generation and analysis of dissociation curves and by 3% agarose gel electrophoresis. The expression of galectin-3 was calculated using the comparative $\left( { 2^{{ - \Delta \Delta C_{\text{q}} }} } \right)$ method. All cell groups were compared against untransfected cells control group at 24 h after the reverse transfection, considered as 100%. The reference gene, chosen for normalization, was *TBP* (*TATA*-*box binding protein*) housekeeping gene. The *Primer*-*BLAST* software (NCBI) was used to designed primers for galectin-3: 5′-GCC AAC GAG CGG AAA ATG G-3′ (forward), 5′-TCC TTG AGG GTT TGG GTT TCC-3′ (reverse); for *TBP*: 5′-TAT AAT CCC AAG CGG TTT GCT G-3′ (forward), 5′-GCC AGT CTG GAC TGT TCT TCA-3′ (reverse). Samples were prepared in triplicate and Real Time™ RT-PCR measurement for each sample was done in duplicate.

### Protein isolation and ELISA assay

Total protein extraction from the cells reverse transfected with RNA oligonucleotides in 24-well plates (24 h, 48 h, and 72 h after reverse transfection) followed by ELISA assay was performed using Galectin-3 Human SimpleStep ELISA^®^ Kit (Abcam) according to the instruction manual. The protein concentration of the samples was determined using Bradford Reagent (Sigma-Aldrich), according to the manufacturer’s protocol, supplemented with Bovine Serum Albumin Standard Set (Fermentas). The absorbance of each sample was measured with ELISA plate reader (Dynex Technologies Triad Multi-Mode Microplate Reader) at 595 nm (Bradford assay) or 450 nm (ELISA assay). In each sample, the concentration of galectin-3 in 1 µg of total protein extract was calculated by interpolating the blank control subtracted absorbance values against the standard curve, followed by multiplying the resulting value by dilution factor. Samples were prepared in triplicate and absorbance measurement for each sample was done in duplicate.

### Cell viability assay

To performed cell viability assay, the cells were reverse transfected with RNA oligonucleotides in 96-well plates and treated with cisplatin for 24 h at 48 h after the reverse transfection, exactly as described above (section: cell culture, reverse transfection, and apoptosis induction). Following the cisplatin treatment (72 h after reverse transfection), the medium in each well was aspirated and replaced with 100 µl of the mixture containing: RPMI-1640 medium without phenol red (Gibco^®^); XTT solution (BioShop Canada Inc; dissolved in PBS from Gibco^®^) at the final concentration of 200 µg/ml and PMS (phenazine methosulfate) solution (Sigma-Aldrich; dissolved in PBS from Gibco^®^) at the final concentration of 2 µg/ml. After the incubation at 37 °C for 3 h in the dark, the absorbance of each well was measured with ELISA plate reader (Dynex Technologies Triad Multi-Mode Microplate Reader) at 450 nm. All cell groups were compared against untransfected and cisplatin-untreated cells’ control group (considered as 100% viable). Samples were prepared in triplicate.

### Cell proliferation assay

For the proliferation assay, the cells were reverse transfected with RNA oligonucleotides in 96-well plates and treated with cisplatin for 24 h at 48 h after the reverse transfection, exactly as described above (section: cell culture, reverse transfection, and apoptosis induction). Following the cisplatin treatment (72 h after reverse transfection), it was performed the EdU (5-ethynyl-2′-deoxyuridine) incorporation assay using Click-iT^®^ EdU Imaging Kit (Invitrogen™) according to the instruction manual, and also the nuclei visualization by staining for 5 min in DAPI (4′,6-Diamidino-2-phenylindole dihydrochloride; Sigma-Aldrich) at the final concentration of 5 µg/ml. The cells were analyzed using an Eclipse Ti (Nikon Instruments Inc.) fluorescence microscope. The ratio of proliferating cells (EdU positive) to the total number of cells (DAPI positive), in three different fields, was calculated for each well. All cell groups were compared against untransfected and cisplatin-untreated cells control group (considered as 100% proliferating). Samples were prepared in triplicate.

### Apoptosis assay

The apoptosis assay was performed using Cell Death Detection ELISA^PLUS^ (Roche), which is an ELISA assay for the quantitative determination of cytoplasmic histone-associated DNA fragments (mono- and oligonucleosomes). The cells were reverse transfected with RNA oligonucleotides in 96-well plates and treated with cisplatin for 24 h at 48 h after the reverse transfection, exactly as described above (section: cell culture, reverse transfection, and apoptosis induction). Following the cisplatin treatment (72 h after reverse transfection), the procedure was carried out according to the manufacturer’s protocol and the absorbance of each well was measured with ELISA plate reader (Dynex Technologies Triad Multi-Mode Microplate Reader) at 405 nm. For each group, the average background value was subtracted from the average absorbance measurement, followed by the calculation of the enrichment factor. The factor represented the ratio of each cells group to untransfected and cisplatin-untreated cells’ control group. Samples were prepared in triplicate and absorbance measurement for each sample was done in duplicate.

### Statistical analysis

*Statistica 12.5* (StatSoft) was used to test differences between the groups by a two-way analysis of variance (ANOVA) followed by post hoc Tukey’s HSD test. Shapiro–Wilk test and Levene’s test were utilized to check the assumptions of normal distribution of variables and homogeneity of variances in each group, respectively. A *p* values less than 0.05 were considered as significant. Data are presented as the mean ± standard deviation (SD).

## Results

### The efficiency of reverse transfection of SK-OV-3 and TOV-21G ovarian cancer cells

**Fig. 1:**
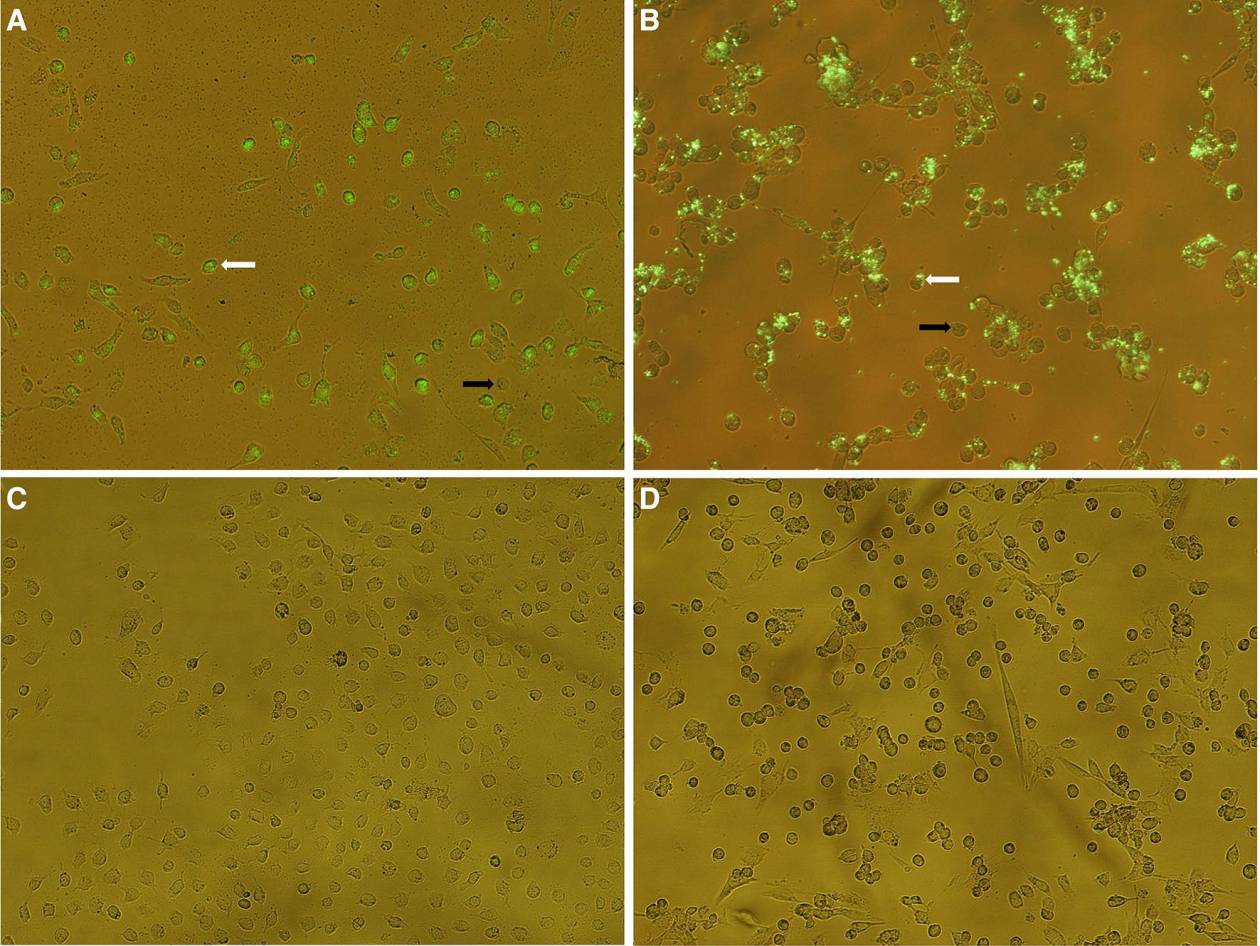
Human ovarian cancer cells reverse transfected with 5′-FAM-labeled miR-424-3p mimic (**a** SK-OV-3; **b** TOV-21G) or the negative control (**c** SK-OV-3; **d** TOV-21G). Images were captured at ×100 magnification by an Eclipse Ti (Nikon Instruments Inc.) fluorescence microscope. **a** and **b** Presence of green fluorescence indicated successfully transfected cells (exemplary transfected cells are marked with white arrows and untransfected cells with black arrows). The efficiency of reverse transfection (the ratio of cells successfully transfected with 5′-FAM-labeled miR-424-3p mimic to the total number of cells) was approximately 97% in both cell lines

First of all, we estimated the reverse transfection efficiency to be approximately 97% in SK-OV-3 and TOV-21G ovarian cancer cell lines based on the ratio of cells successfully transfected with 5′-FAM-labeled miR-424-3p mimic (the presence of green fluorescence) to the total number of cells in three different fields. Figure 1 shows an exemplary image of SK-OV-3 (Fig. 1a, c) and TOV-21G cells (Fig. 1b, d) captured by an Eclipse Ti (Nikon Instruments Inc.) fluorescence microscope.

**Fig. 2:**
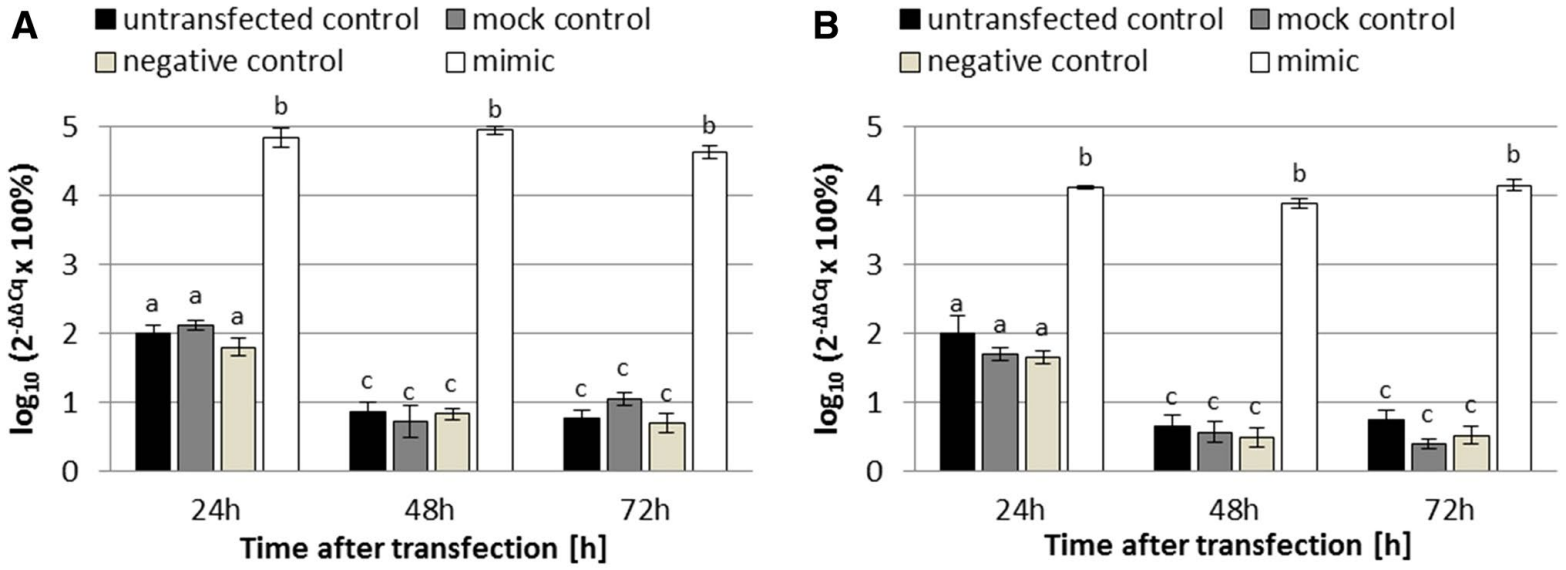
Expression of miR-424-3p in SK-OV-3 (**a**) and TOV-21G (**b**) human ovarian cancer cells at 24 h, 48 h, and 72 h after the reverse transfection of miR-424-3p mimic into both cell lines. The expression was analyzed by the Real Time™ stem-loop RT-PCR technique ($2^{{ - \Delta \Delta C_{\text{q}} }}$ method). The data are shown as mean ± SD of triplicate experiments. A two-way analysis of variance (ANOVA), followed by post hoc Tukey’s HSD test, was used to test statistical differences between the studied groups. *p* values less than 0.05 were considered as significant. All bars on the charts, which are marked with the same letter (even if it is only one common letter among many), are not significantly different

Second of all, we confirmed the reverse transfection effectiveness by Real Time™ (stem-loop) RT-PCR analysis. The Real Time™ RT-PCR instrument did not detect any amplification (no Cq values) in no template controls. Figure 2 shows a statistically significant increase in miR-424-3p expression (24 h, 48 h, and 72 h) after the reverse transfection of the miR-424-3p mimic into both cell lines. In SK-OV-3, 24 h after the reverse transfection, the amount of miR-424-3p was approximately 1000-fold greater in the mimic group compared to all control groups (all Tukey’s HSD *p* < 0.001; Fig. 2a) and it continued to be at the same level for 48 h and 72 h after the reverse transfection (all Tukey’s HSD *p* > 0.05; Fig. 2a). Similarly, at 24 h after the reverse transfection of TOV-21G, the expression of miR-424-3p was about 100-fold higher in the mimic group compared to all control groups (all Tukey’s HSD *p* < 0.001; Fig. 2b) and it remained at the same level for 48 h and 72 h after the reverse transfection (all Tukey’s HSD *p* > 0.05; Fig. 2b).

### The effect of miR-424-3p on expression of galectin-3 in SK-OV-3 and TOV-21G ovarian cancer cells

**Fig. 3:**
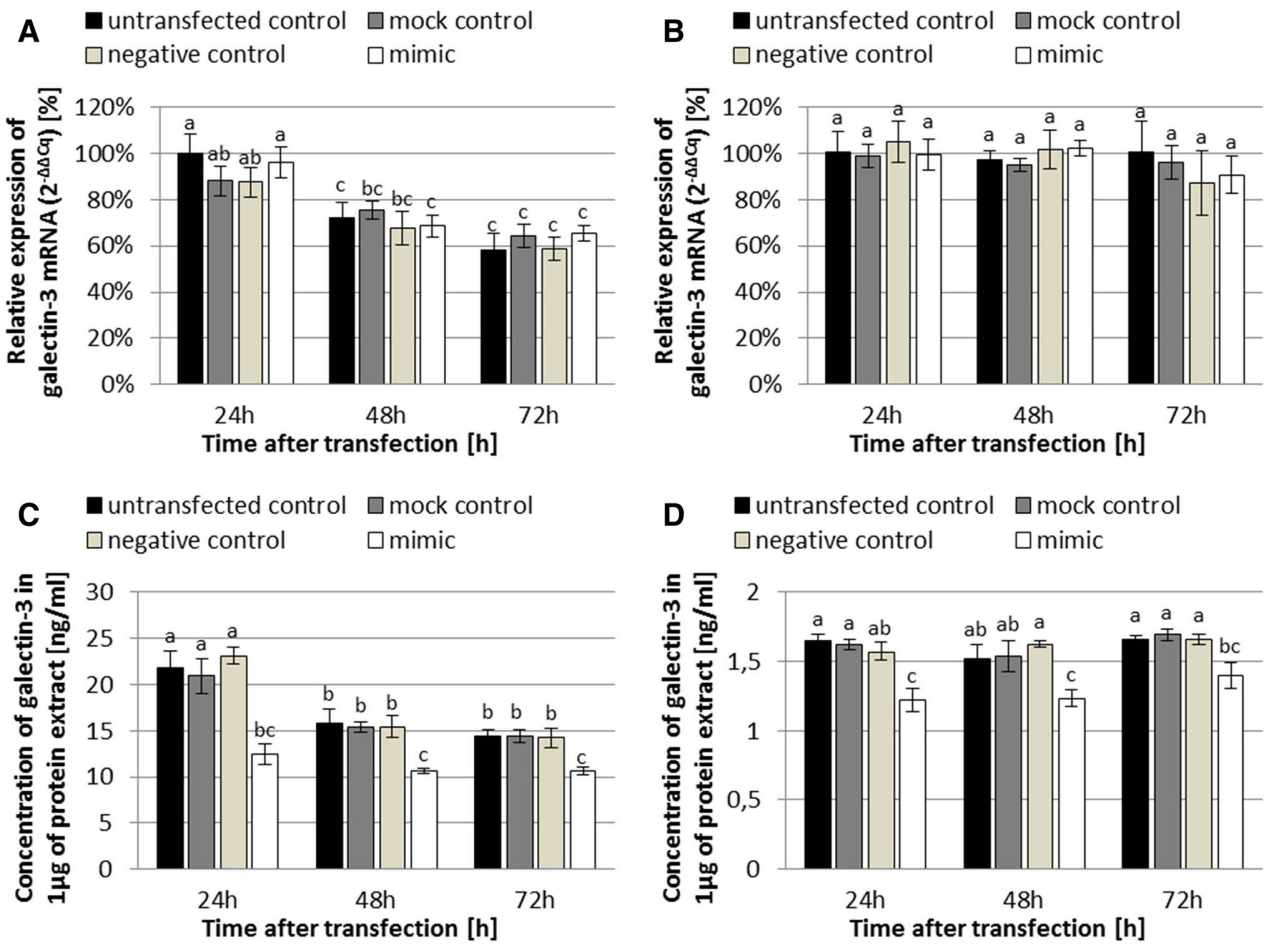
Expression of galectin-3 at the mRNA and protein levels in SK-OV-3 (**a** mRNA; **c** protein) and TOV-21G (**b** mRNA; **d** protein) human ovarian cancer cells at 24 h, 48 h, and 72 h after the reverse transfection. The expression was analyzed by Real Time™ RT-PCR technique at the mRNA level ($2^{{ - \Delta \Delta C_{\text{q}} }}$ method) and by ELISA assay at the protein level. The data are shown as mean ± SD of triplicate experiments. A two-way analysis of variance (ANOVA), followed by post hoc Tukey’s HSD test, were used to test statistical differences between the studied groups. A *p* values less than 0.05 were considered as significant. All bars on the charts, which are marked with the same letter (even if it is only one common letter among many), are not significantly different

To investigate the effect of miR-424-3p on the expression of galectin-3, we performed Real Time™ RT-PCR analysis and ELISA assay on the RNA and protein extracts obtained from SK-OV-3 and TOV-21G ovarian cancer cells, which we had previously transfected with miR-424-3p mimic. The instrument, which was proceeding Real Time™ RT-PCR analysis, did not register any amplification (no Cq values) in no template controls. Standard curves generated in Bradford and ELISA assays had the *r*^2^ values greater than 0.991 and 0.999, respectively. The results are shown in Fig. 3.

At the mRNA level, there were no statistical changes due to the reverse transfection in both cell lines. In each time period after the reverse transfection, the expression of galectin-3 in each mimic group did not significantly differ in comparison to each relevant (untransfected, mock, and negative) control group (all Tukey’s HSD *p* > 0.05; Fig. 3a and b). On the other hand, at the protein level occurred a statistically significant reduction in galectin-3 expression (a decrease of approximately 50% in SK-OV-3 and 25% in TOV-21G) between all mimic groups and all related to them control groups in each time period after the reverse transfection and in both cell lines (all Tukey’s HSD *p* < 0.001 except the four *p* < 0.05 for TOV-21G at 72 h; Fig. 3c and d).

Surprisingly, the results also revealed that the amount of galectin-3 mRNA and protein in SK-OV-3 cells statistically decreased during the time of the experiment (when comparing the same type of group between all three time periods; all Tukey’s HSD *p* < 0.05; Fig. 3a and c), but this was not observed in TOV-21G cells (all Tukey’s HSD *p* > 0.05; Fig. 3b and d).

### The influence of miR-424-3p on sensitivity of SK-OV-3 and TOV-21G ovarian cancer cells to cisplatin

**Fig. 4:**
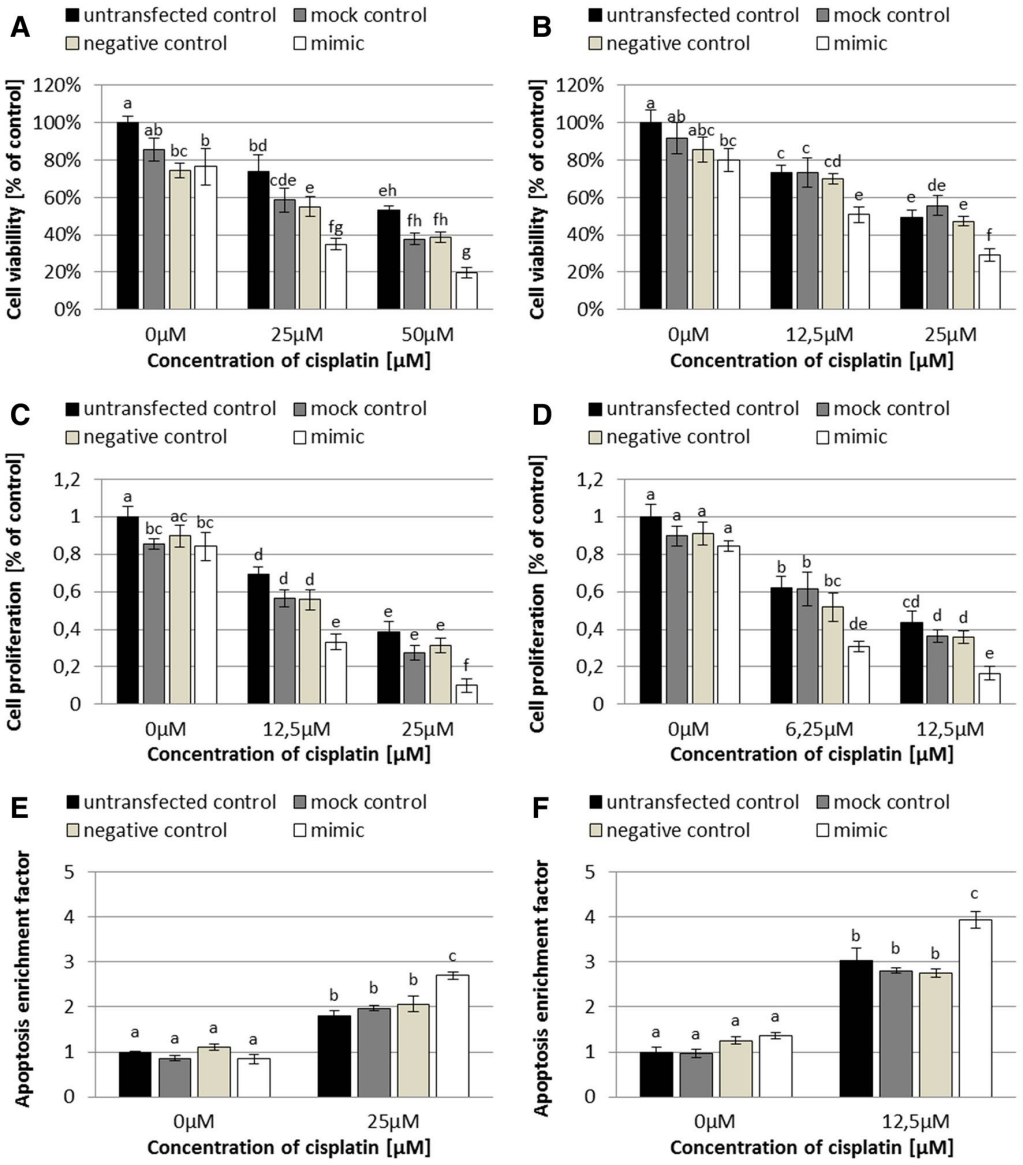
Analysis of human ovarian cancer cells: viability by XTT assay (**a** SK-OV-3; **b** TOV-21G); proliferation by EdU incorporation assay (**c** SK-OV-3; **d** TOV-21G); apoptosis by quantitative determination of cytoplasmic histone-associated-DNA-fragments in ELISA assay, followed by the calculation of enrichment factor for each studied group (**e** SK-OV-3; **f** TOV-21G). The cells were reverse transfected and treated with cisplatin for 24 h, at 48 h after the reverse transfection. The data are shown as mean ± SD of triplicate experiments. A two-way analysis of variance (ANOVA), followed by post hoc Tukey’s HSD test, were used to test statistical differences between the studied groups. A *p* values less than 0.05 were considered as significant. All bars on the charts, which are marked with the same letter (even if it is only one common letter among many), are not significantly different

To study the influence of miR-424-3p on sensitivity of SK-OV-3 and TOV-21G ovarian cancer cells to cisplatin, we performed cell viability (XTT), proliferation (EdU incorporation), and apoptosis assays on the cells reverse transfected and (after 48 h) treated with cisplatin for 24 h to induce apoptosis. The concentrations of cisplatin used during the experiments were selected based on the cisplatin response curves as well as IC_50_ (half maximal inhibitory concentration) and GI_50_ (half maximal growth inhibitory concentration) values obtained for TOV21-G and SK-OV-3 cells in our previous studies [19]. The results are shown in Fig. 4. Viability, proliferation, and apoptosis of SK-OV-3 and TOV-21G cells untreated with cisplatin were essentially comparable (between relevant control groups and mimic group). However, cisplatin treatment revealed statistically significant differences between mimic groups and related to them (untransfected, mock, and negative) control groups in both cell lines (all Tukey’s HSD *p* < 0.01 in XTT assay and < 0.001 in EdU incorporation and apoptosis assays).

## Discussion

Ovarian cancer is one of the most deadly gynecological cancers (the fifth leading cause of cancer death among women worldwide), due to the difficulties in the early detection and the development of chemotherapy resistance. Approximately 20% of all primary ovarian cancer patients are resistant to the standard platinum-based chemotherapy, and the remaining 80%, initially responsive to therapy, finally, experience relapse with the acquired drug resistance, which ultimately leads to death. Therefore, overcoming the resistance is one of the most critical challenges in ovarian cancer therapy [1–3]. Cisplatin, introduced to clinical trials in 1971, is still a successful anticancer drug and it is widely used in the treatment of solid tumors, such as ovarian, breast, head and neck, and lung cancers. In the clinical practice, cisplatin is used in combination with other chemotherapeutic agents (e.g., with paclitaxel), because cisplatin alone often results in chemoresistance [2, 11]. On the other hand, a better understanding of the etiology of ovarian cancer has led to the progression of molecular targeted therapies. Many small molecules, which target critical cancer traits, are the subjects of multiply studies. Among these molecules are miRNAs (small, non-coding RNA molecules of 20–22 nucleotides in length), which are capable to regulate gene expression and are involved in ovarian cancer tumorigenesis [1, 2]. An interesting potential target for miRNAs could be galectin-3, since it is an anti-apoptotic protein that is overexpressed in various tumor cells (including ovarian cancer) and involved in their chemoresistance [3, 5, 7]. Moreover, it has been proved that miR-424-3p (previously also known as miR-322) can modulate galectin-3 expression by decreasing its level [12]. Furthermore, miR-424-3p has been also proved to prevent proliferation, migration, and invasion of non-small-cell lung cancer cells as well as sensitize them to cisplatin and paclitaxel by inhibiting YAP1 protein [13]. However, until now, there has been no research on miR-424-3p in ovarian cancer. In the present study, we, for the first time, demonstrate, that miR-424-3p mimic can suppress galectin-3 expression in ovarian cancer cells, and thus sensitize them to cisplatin.

First of all, we performed a reverse transfection of SK-OV-3 (cisplatin-resistant) and TOV-21G (cisplatin-sensitive) ovarian cancer cells with an efficiency of approximately 97% (Fig. 1), which is a comparable value to the other studies with miRNA mimics [20]. We also confirmed the effectiveness of the reverse transfection by the Real Time™ PCR analysis. In cells transfected with miR-424-3p mimic, we noticed approximately 1000-fold (SK-OV-3) and 100-fold (TOV-21G) increase of miR-424-3p expression compared to control groups (all Tukey’s HSD *p* < 0.001; Fig. 2). Moreover, the upregulation of miR-424-3p was stable for 72 h after the reverse transfection (Fig. 2). Furthermore, the cell viability assay demonstrated that cytotoxicity (about 15% in SK-OV-3 and 10% in TOV-21G) of the used concentration of Lipofectamine^®^ 2000 Reagent was not statistically significant (untransfected control groups vs mock control groups) in both cell lines (both Tukey’s HSD *p* > 0.05; Fig. 4a, b). Therefore, these results indicate that we have established efficient, effective and cell-safe conditions for the reverse transfection of SK-OV-3 and TOV-21G cell lines.

Second of all, we evaluated the effect of miR-424-3p mimic on the expression of galectin-3 in SK-OV-3 and TOV-21G ovarian cancer cells. Galectin-3, a β-galactoside binding lectin, is a well-known suppressor of apoptosis. Chemotherapeutic agents, such as cisplatin, cause the phosphorylation and relocation of the protein from the nucleus to the cytoplasm, where it stabilizes BCL-2, thus blocks cytochrome c release, and inhibits apoptosis [7, 11]. The key element of galectin-3 structure, allowing to suppress apoptosis, is an NWGR (Asp–Trp–Gly–Arg) anti-death motif inside BH1 domain, which galectin-3 shares with the BCL-2 family of proteins [5]. Numerous studies indicate that galectin-3 is involved in chemoresistance of various tumors, such as ovarian [3, 21], pancreatic [6], colorectal [9], thyroid [22] and prostate cancer [23], as well as osteosarcoma [10] and cholangiocarcinoma [24] and that the reduction of its expression sensitizes these cancer cells to chemotherapeutic agents.

Our data showed that miR-424-3p mimic decreased the expression of galectin-3 (by approximately 50% in SK-OV-3 and 25% in TOV-21G), but only at the protein level (all Tukey’s HSD *p* < 0.05; Fig. 3c and d), without affecting the mRNA level (all Tukey’s HSD *p* > 0.05; Fig. 3a and b). It seems that miR-424-3p acts only through inhibition of galectin-3 translation without degradation of the mRNA, which is one of the possible mechanisms of action of miRNA described in the literature [25, 26]. Moreover, another miRNA molecule targeting galectin-3 (miR-128) also successfully downregulated galectin-3 protein levels in HT29 and SW620 colorectal cancer cell lines, but not mRNA levels [9]. On the other hand, Ramasamy et al. [12] obtained conflicting results, in which miR-424-3p reduced both mRNA (by about 50%) and protein levels of galectin-3 in BT549 breast cancer cells. Our results also surprisingly revealed that the expression of galectin-3 mRNA and protein in SK-OV-3 cells statistically decreased at 48 h and 72 h after the reverse transfection (all Tukey’s HSD *p* < 0.05; Fig. 3a and c), but this was not observed in TOV-21G cells (all Tukey’s HSD *p* > 0.05; Fig. 3b and d). We believe that it was caused by serum starvation implemented at 24 h after the reverse transfection, since similar cases are described in the literature [27].

Third of all, we studied the influence of miR-424-3p mimic on sensitivity of SK-OV-3 and TOV-21G ovarian cancer cells to cisplatin. We found that the viability and proliferation of both cell lines, reverse transfected with miR-424-3p mimic and treated with cisplatin, were statistically decreased (all Tukey’s HSD *p* < 0.01 in viability assay and < 0.001 in proliferation assay; Fig. 4a–d) and similarly, apoptosis of these cells was significantly induced (all Tukey’s HSD *p* < 0.001; Fig. 4e and f). However, in case of both of these cell lines reverse transfected with miR-424-3p, but untreated with cisplatin, generally, there were no differences (comparison of relevant control groups and mimic group) in viability, proliferation, and apoptosis (all Tukey’s HSD *p* > 0.05; Fig. 4). Our results are partially consistent with the published research. Similar to our data, downregulation of galectin-3 by siRNA in ovarian clear cell carcinoma [21] and osteosarcoma (to approximately 33%) [10] did not affect proliferation and apoptosis of cancer cells; however, in combination with cisplatin, it increased the extent of apoptosis [10, 21] and inhibited proliferation [21]. Moreover, galectin-3 silencing alone by siRNA also had no effect on pancreatic cancer cell proliferation, until cisplatin treatment was implemented at 48 h after transfection. However, on the contrary to our data, galectin-3 silencing alone by siRNA was sufficient to induce apoptosis of the pancreatic cancer cells [6] as well as to inhibit proliferation of SK-OV-3 and OVCRA429 ovarian cancer cells [3]. On the other hand, the sensitivity of cholangiocarcinoma cells to cisplatin- and 5-FU-induced apoptosis was increased only when galectin-3 expression was almost completely diminished by siRNA (< 10%), whereas the reduction of galectin-3 expression only to 50% did not induce apoptosis [24]. Moreover, not only siRNAs but also another miRNA molecule (miR-128) was able to sensitize HT-29 and SW620 colorectal cancer cells to chemotherapy and inhibit invasion potential these cells by decreasing the level of galectin-3 [9].

To briefly summarize, we believe that we demonstrated the potential of miR-424-3p to sensitize of ovarian cancer cells to cisplatin by decreasing galectin-3 expression. Even though improvements in platinum-based chemotherapy have been made, it still has a relative low response rate, and if it occurs the chemoresistance, then no alternative treatment is available. On the other hand, miRNAs have been shown to affect multiply cellular functions, including proliferation, differentiation, cell cycle, and apoptosis, and thus, they are associated with various kinds of cancers [2]. Each miRNA controls the expression of hundreds of different genes and can effectively control multiple cellular pathways. Therefore, miRNAs have been offered as possible therapeutic weapons against cancer. Since MRX34 (the first ‘anticancer miRNA drug’, which is a miR-34 mimic) has entered clinical trials in patients with advanced hepatocellular carcinoma, the possibility of including miRNAs for the cancer treatments might become much more possible in the near future and with the progression in cancer research, therapies may soon be customized for each individual [1].

## Conclusion

In this paper, we demonstrated that miR-424-3p mimic sensitizes TOV-21G (cisplatin-sensitive) and SK-OV-3 (cisplatin-resistant) ovarian cancer cells to cisplatin by decreasing the expression of galectin-3 (an anti-apoptotic protein). Our findings could make miR-424-3p a useful candidate for oncological combination treatment with cisplatin.
